# Copper Nanoparticles in Aquatic Environment: Release Routes and Oxidative Stress-Mediated Mechanisms of Toxicity to Fish in Various Life Stages and Future Risks

**DOI:** 10.3390/cimb47060472

**Published:** 2025-06-19

**Authors:** Anna Sielska, Lidia Skuza

**Affiliations:** 1 Institute of Biology, University of Szczecin, PL-71-415 Szczecin, Poland; 2 Centre for Molecular Biology and Biotechnology, University of Szczecin, PL-71-415 Szczecin, Poland

**Keywords:** copper nanoparticles, farmed fish, ecotoxicology, aquatic environment

## Abstract

The final recipient of nanoparticles, including various types of copper-based nanoparticles (Cu-based NPs), is the aquatic environment. Their increased production, especially as a component of antimicrobial agents, raises concerns about uncontrolled environmental release and subsequent ecological risks. The high reactivity of Cu-based NPs enables interactions with biotic and abiotic environmental components, leading to bioaccumulation and disorders in living organisms, such as fish in various life stages, especially in embryos or hatchlings. Increasing concentration of Cu-based NPs causes various toxic effects, mainly through the induction of oxidative stress. These effects include impairment of antioxidant mechanisms, as well as damage to genetic material, cells and tissues, growth retardation, metabolic disorders, increased mortality, or hatching inhibition. The aim of this review is to describe the release routes of Cu-based NPs and their adverse effects on fish, while emphasizing the need for further research on their toxicity and measures to control their release to the environment. Given the limited data on the toxicity of Cu-based NPs, especially concerning sensitive fish developmental stages, further studies are required.

## 1. Introduction

The European Union has collectively defined all nanoparticles under the term nanomaterials as “a natural, incidental, or manufactured material consisting of solid particles, either in an unbound state or as identifiable constituent particles in aggregates or agglomerates, where at least 50% of these particles in the numerical size distribution meet at least one of the following conditions:

(a) At least one external dimension of the particle is in the size range of 1–100 nm.

(b) The particle has an elongated shape, such as a rod, fiber, or tube, where two external dimensions are smaller than 1 nm and the other dimension is larger than 100 nm.

(c) The particle has a plate-like shape, where one external dimension is smaller than 1 nm, and the other dimensions are greater than 100 nm” [1]. However, the International Organization for Standardization (ISO) has further classified nanomaterials into two distinct categories: nanostructured materials and nano-objects, the latter of which includes nanoparticles [2]. Nanomaterials, including nanoparticles, can form naturally or through anthropogenic activities—synthesized using engineering techniques, chemical methods, or biological systems [3,4]. In addition to naturally occurring and synthetic ones, nanomaterials and nanoelements are classified into several groups [5,6]: carbon-based (carbon nanotubes, graphene), organic (based on organic molecules, including polymers), and inorganic, which may include nanocomposites, non-metallic nanoelements, and metallic nanoelements (silver, gold, and copper nanoparticles). Nanoelements, such as silver, iron compounds, and magnetite, ubiquitous in the environment, are structural components of various biological systems, including insect exoskeletons (chitin), animal bones, and even those synthesized by microorganisms [7,8]. Other natural sources of nanoparticles include deserts, as well as phenomena such as volcanic eruptions and wildfires [3,9].

Engineered nanoparticles (ENPs) are molecules produced through chemical synthesis or biological systems. They typically include metal nanoparticles, as well as nanomaterials such as nanocomposites and carbon nanotubes [5]. ENPs are used in the production of plastics, textile coatings, fertilizers, and medical products.

In addition to their small size, nanoparticles are characterized by a variety of shapes. Moreover, nanoparticles can form structures such as nanotubes and micelles, and their surface can be functionalized with other molecules, including amino acids, lipids, sugars, and chemical groups [10]. These characteristics give them unique physicochemical and biological properties not found in other forms of heavy metals [11,12]. As a result, synthetic nanoparticles, particularly those produced through “green” synthesis methods, represent an attractive potential alternative to heavy metals currently used in industries, which are generally viewed as environmental pollutants with strong toxic effects on living organisms, including fish [13,14]. Due to their increased production, nanoparticles, including Cu-NPs, are now also classified as environmental pollutants that adversely affect both wild and farmed fish species. Salmonids (*Salmonidae*) are considered particularly sensitive to environmental changes such as climate warming and pollution, which affect their growth and development [15,16]. Similar susceptibility to environmental toxins and contaminations has been documented in *Cyprinus carpio* [17,18]. Moreover, due to the widespread farming of *C. carpio*, *Salmo trutta,* and *Oncorhynchus mykiss*, particularly for consumption and food production, there is a clear need for research on the impact of environmental pollutants and toxins on these species. The literature data suggest that, in addition to the model species *Danio rerio*, farmed species such as *C. carpio* and *O. mykiss* can be used in studies on the detection of heavy metal pollution in aquatic environments (scales, sperm, metallothionein levels), serving as suitable model species in ecotoxicological research [19,20,21].

The aim of this review article was to present the problem of the negative impact of Cu-based NPs on fish resulting from their uncontrolled release into the environment. Consequently, this review describes Cu-based NPs and their toxicity mechanisms related to oxidative stress induction, while also proposing mitigation measures to limit Cu-based NPs emissions, particularly into aquatic environments.

## 2. Copper Nanoparticles

Copper-based nanoparticles (Cu-based NPs), due to their unique characteristics, serve multiple industrial purposes. Their enhanced optical properties make them suitable for applications in photocatalysis, photovoltaics, and as components of conductive inks [22,23,24]. Additionally, nanocomposites containing copper nanoparticles exhibit high thermal and electrical conductivity [25]. The exceptional optical, thermal, and electrical properties position Cu-based NPs as highly promising semiconductor materials [26]. Nanoparticles are also used in agriculture, including as components of fertilizers [27,28] and pesticides [29,30]. However, the most desirable feature of Cu-based NPs is their exceptional biocidal properties, which is why they are primarily used in medicine. Research has demonstrated that copper nanoparticles (Cu-NPs) exhibit strong strain-specific antibacterial activity, often comparable to silver nanoparticles (Ag-NPs), particularly against bacterial strains like *Staphylococcus aureus* [31,32]. Vasiliev et al. (2023) further highlighted enhanced antibacterial performance through synergistic interactions between these metallic nanoparticles [33]. Consequently, copper nanoparticles can be used to coat textiles, particularly in hospital settings, to impart biocidal properties, protecting both patients and staff from infections [34]. Moreover, Cu-NPs demonstrate notable anticancer properties and have emerged as promising drug delivery carriers, offering potential for use in cancer therapies [35,36,37].

## 3. Sources of Copper Nanoparticles and Their Interactions with Aquatic Environments

The high potential of Cu-based NPs and their numerous applications have contributed to their widespread use and increased production. Products containing nanoparticles (NPs) are present in almost every aspect of human life. The issue, however, arises from the uncontrolled release of nanoparticles into the environment, as they are considered pollutants in this form [38,39]. Their sources include industrial waste, municipal waste, medical waste, and household products [40]. Nanoparticles, occurring in various forms such as aerosols, colloids, solid particles, or as components of solutions, enter all environmental spheres (air, water bodies, and soil) through multiple routes, including wastewater, runoff from agricultural fields, and factory emissions [40,41]. Ultimately, all pollutants associated with nanoparticles reach their final receptor, which is the aquatic environment [42,43]. Nanoparticles present in aquatic environments undergo diverse transformations and interact with dissolved molecules, organic/inorganic matter, and living organisms. Tierney and Pyle (2024) raised concerns that aquatic pollution from metals and their nanoparticles may adversely affect salmonids, including through sensory disruptions (impaired chemosensory perception), which contribute to abnormal migratory behaviors [44]. The intrinsic properties of nanoparticles (e.g., shape, size, surface charge, and ligand presence), combined with environmental factors (temperature, salinity, water hardness, and pH), enable diverse aquatic interactions: biological (biodegradation, organic matter interactions, or bioadsorption), chemical (ion release and sulfidation), and physical (aggregation and adsorption) [45,46,47]. Thus, in aquatic reservoirs, nanoparticles exist in various forms (aggregates, ions, or complexes) distributed throughout the water column, surface layers, and benthic sediments [48]. Nanoparticle behavior differs significantly between freshwater and saline environments. Conway et al. (2015) demonstrated that in the presence of ions characteristic of marine systems (phosphates, chlorides, or sulfates), Cu-NPs form insoluble complexes, both with inorganic ions and organic matter [49]. Studies have also shown that Cu-NPs exhibit increased agglomeration tendencies with rising pH levels [50]. Xiao et al. (2018), using *Daphnia magna* as a model organism, demonstrated that changes in water chemistry parameters significantly influence toxicity to aquatic organisms [51].

The presence of nanoparticles in the environment leads to bioaccumulation by living organisms, which can occur through both direct and indirect pathways. Cu-NPs enter organisms directly by endocytosis and, when present as ions or very small particles, via gill diffusion [52]. Studies confirm that nanoparticle size and surface ligands significantly influence cellular internalization through endocytosis or pinocytosis, as well as their biological interactions and systemic behavior [53]. Cu-NPs are bioaccumulated indirectly through trophic transfer [54]. When nanoparticles form aggregates, they may settle and deposit on the bottom or in benthic sediments, where they are ingested by organisms, or on plant surfaces, which are consumed by herbivorous species. They can also be absorbed and accumulated by algae or invertebrates [55,56,57,58], potentially leading to their transfer through food chains to higher trophic levels, including humans [59]. Yu et al. (2022) observed that Cu-NP uptake in aquatic organisms occurred primarily through dietary exposure [60], while Wu et al. (2017) proposed that the combination of direct and indirect pathways may increase the toxicity of these nanoparticles [54].

## 4. Concentrations and Monitoring of Cu-NPs in Aquatic Environments

Currently, data on hazardous concentrations of Cu-based NPs in the environment are lacking, and studies on their toxicity remain scarce. Current knowledge regarding copper-based nanoparticle concentrations in the environment remains limited [61]. Barreto et al. (2021) used a probable natural Cu-NPs concentration range of 0.3–635 µg/L in their studies on *Ankistrodesmus densus* algae [62], while Xu et al. (2020) reported a concentration of those nanoparticles of approximately 0.79 × 10^7^ particles/mL in Jiaozhou Bay’s surface waters [63]. Earlier work by Chio et al. (2012) estimated a general environmental concentration of Cu-NPs at approximately 60 μg/L [64]. Although these presented values remain relatively low, researchers caution about potential gradual increases in the coming years [61,62]. Notably, some regions may already experience high localized Cu-NP concentrations that remain undocumented.

Current low environmental concentrations and potentially high costs [65] have prevented the establishment of monitoring programs for copper nanoparticles in the environment. Growing concerns about projected increases in Cu-NP concentrations have intensified calls for implementing comprehensive environmental monitoring programs [61,66]. Consequently, various monitoring methods are being proposed and described in the literature. Wu et al. (2020), in their study on *Chlamydomonas reinhardtii*, *Escherichia coli*, *Daphnia magna*, and *Danio* *rerio* embryos, proposed using multi-species small-scale microcosm/nanocosm analysis for rapid assessment of NP toxicity [65]. This method evaluates toxic relationships across trophic levels and environmental species [65]. Their analysis included accumulation rates in different organisms and toxicity parameters (algal growth inhibition, *D. magna* survival, and *D. rerio* hatch delay). Using meta-analysis, Parsai et al. (2021) identified three nanoparticle types posing the highest environmental risk, including copper oxide nanoparticles (CuO-NPs), and established their maximum permissible concentrations: 70.8 mg/L for CuO-NPs alone, and 24.7 mg/L or 175.12 mg/L for combined ZnO + CuO NPs aggregates [67]. These authors also found that Ag-NPs, titanium dioxide nanoparticles (TiO_2_-NPs), and CuO-NPs showed the lowest maximum permissible concentration values. Spectroscopic techniques have also been proposed as potential methods for monitoring nanoparticles, such as CuO-NPs, in the environment and their toxicity assessment [68,69]. Given that oxidative stress represents a primary mechanism of Cu-NP toxicity and associated disruptions of antioxidant defense systems, monitoring oxidative stress biomarkers has been proposed as an effective approach for assessing copper nanoparticle levels in aquatic environments [70]. This method focuses on analyzing first-line defense markers against reactive oxygen species (ROS), including key antioxidant enzymes (superoxide dismutase, glutathione peroxidase, catalase) and related genes [71,72,73]. The measurement of *hsp70* gene expression and enzymatic activity in fish serves as a reliable biomarker for assessing water quality and detecting contaminant presence in aquatic ecosystems [74,75]. Additionally, it was reported that heat shock protein *hsp70* gene expression and its enzyme activity can be used as a marker indicating stress induced by nanoparticles, such as Ag-NPs [76] or CuO-NPs [77]. CYP1A also serves as an excellent biomarker for assessing the toxicity of environmental chemical pollutants in fish, including metal nanoparticles [72,78,79,80,81]. Among the cytochrome P450 genes (*cyp*) studied, Cortés-Miranda et al. (2024) identified *cyp1a* as a marker for chronic pollution in fish [82]. Our studies have also confirmed the applicability of these biomarkers in ecotoxicological research on hatchery-reared fish hatchlings [83,84].

## 5. Oxidative Stress-Mediated Toxicity Mechanisms of Cu-Based NPs

The structural properties of Cu-based NPs, particularly their surface characteristics, provide desirable biological and physicochemical features for various industrial applications, but may simultaneously contribute to their toxic potential. Despite the lack of data regarding the toxicity mechanism of Cu-based NPs, the most commonly studied ones are Cu-NPs and CuO-NPs. The structural properties of NPs (shape, size, chemical composition, surface characteristics) and environmental conditions (pH, salinity, temperature) collectively determine Cu-NP solubility and consequently influence their toxicity mechanisms [85] (Table 1). The toxicity of copper nanoparticles and other engineered nanomaterials can be induced through both indirect mechanisms and direct cellular contact, involving interactions with genetic material, organelles, and tissues, ultimately leading to their damage [53]. The induction of oxidative stress represents one of the key mechanisms through which Cu-based NPs exert toxic effects on organisms [86,87]. Naz et al. (2020) have suggested that nanoparticle elements induce toxic effects both indirectly, by triggering the cellular redox system, and directly, where both mechanisms lead to the production of reactive oxygen species (ROS), and thus oxidative stress [88]. Chen et al. (2011) reported that copper (I) oxide nanoparticles (Cu_2_O-NPs) can be internalized inside cells through copper transporters [89]. Other studies suggested that Cu_2_O-NPs cause toxic effects through direct interaction with the cell membrane and ROS generation [90]. Copper sulfide nanoparticles (CuS-NPs) are considered to be less toxic, although their interaction with factors that are present in the environment (e.g., pollutants, such as hypochlorite) can trigger their adverse effects through oxidative stress and cell membrane damage [91]. Li et al. (2015) also reported that the toxicity of CuS-NPs in early life stages of fish (embryos) is mediated by the release of copper ions [92]. Oxidative stress is defined as an imbalance between oxidants, including free radicals, ROS, and antioxidants (such as vitamin C, antioxidant enzymes, and stress-related molecules), leading to cellular damage [93,94,95]. Disruption of this balance contributes to the accumulation of free radicals within cells, resulting in damage to cellular organelles, such as mitochondria [96,97]. Under extreme conditions, free radical concentrations may exceed the detoxifying capacities of antioxidant defense systems [98] and contribute to the induction of apoptosis.

Several models have been proposed to explain oxidative stress triggered by copper-based nanoparticles. Oxidative stress exerts its toxic effects through two primary pathways: (1) direct ROS overproduction and (2) indirect inhibition of antioxidant enzymes, such as glutathione peroxidase and catalase, by overwhelming their protective functions [87,98]. Kodali and Thrall (2015) have also suggested that the generation of free radicals by nanomaterials occurs through the modification of cellular metabolism and gene expression, with the most common mechanism being the disruption of cellular respiration in mitochondria [99]. Copper nanoparticles can generate free radicals and ROS through the so-called “Trojan horse mechanism”, a process that also shows potential for medical applications in anticancer therapies [100]. This mechanism exploits key nanoparticle properties, particularly their small size and molecular structure, which enable targeted cellular delivery [101,102]. In this model, copper nanoparticles act as molecular carriers, transporting ions and other cargo into cells via the endosomal-lysosomal pathway [100,103]. Upon reaching lysosomes, the acidic environment dissolves Cu-NPs, triggering massive metal ion release and subsequent free radical generation [104,105].

Another oxidative stress induction mechanism involves the Fenton reaction, which can also be associated with the Trojan horse mechanism, utilizing the presence of significant quantities of ions released through this process [105,106]. The Fenton reaction, commonly involving iron ions [107], can also occur with metals such as copper and copper-based nanoparticles, such as CuO-NPs (Fenton-like reactions) [106,108]. It is a two-step process during which Cu^+^ is oxidized in the presence of hydrogen peroxide (H_2_O_2_), resulting in the formation of Cu^2+^ ions, hydroxide anions, and highly reactive hydroxyl radicals. In the second stage, the Haber–Weiss reaction occurs, where Cu^2+^ is reduced to Cu^+^ in the presence of superoxide anion and hydrogen peroxide, enabling the Fenton reaction to proceed again [106,109]. Kessler et al. (2022) stated that metallic Cu-NPs generate high concentrations of ROS through Fenton-like reactions that occur on their surface [106].

**Table 1:** Toxicity of selected copper-based nanoparticles. Adverse effects marked in bold represent oxidative stress-mediated disorders.

Nanoparticle Type	Species	Adverse Effects	Source
Cu-NPs	*Pangasianodon hypopthalmus*	Acetylcholinesterase inhibition	[110]
	*Danio rerio*	Intestinal microvilli damages, **Intestinal damages** **Oxidative stress markers** **Downregulation** **Mitochondrial damages** Endoplasmic reticulum stress marker upregulation	[111]
CuO-NPs	A549 cell line	**Cell cycle arrest (phase S)**	[97]
	*Cyprinus carpio*	Nucleus damages	[112]
Cu_2_O-NPs	A549 cell line	Mitochondrial fragmentation	[97]
	*Danio rerio*/ZFL *Danio rerio liver cell line*	**Oxidative stress-related gene upregulation (ZFL cell line)**, copper transporters induction	[89]
	*Carassius auratus*	Hemolysis, **Red blood cell membrane damages**	[90]
CuS-NPs	*Danio rerio* (embryo)	Embryo coagulation, elevation of MT levels, **lipid peroxidation, antioxidant system depletion** (with hypochlorite)	[91]
	*Oryzias latipes*	**Length reduction**	[92]

## 6. Cu-Based NP Toxicity in Fish Mediated by Oxidative Stress

Numerous studies documented the toxicity of copper nanoparticles, demonstrating genotoxic, cytotoxic, immunotoxic, and organotoxic effects mediated primarily through oxidative stress mechanisms (Table 2 ). However, these analyses have focused exclusively on adult organisms, particularly internal organs of commonly farmed fish species like *S. trutta*, *C. carpio*, or *O. mykiss*. Studies on Cu-based NP toxicity to fish hatchlings remain limited. This review article focuses exclusively on the adverse effects of Cu-based NPs, such as Cu-NPs and CuO-NPs, mediated by oxidative stress. All adverse effects of those nanoparticles are summarized in Table 2, Table 3 and Table 4.

### 6.1. Cytotoxicity and Histopathological Effects of Copper Nanoparticles

Studies have reported cellular organelle damage, including mitochondrial impairment, induced by copper nanoparticles (Table 2). Braz-Mota et al. (2018) observed increased ROS levels in *Apistogramma agassizii* gills due to proton leakage from mitochondria following exposure to CuO-NPs at a dose of 3 mg dm^−3^ [113]. Wang et al. (2015) additionally demonstrated that copper nanoparticles induced mitochondrial membrane damage, enhanced membrane permeability, and reduced metabolic capacity [113,114]. Furthermore, in the PLHC-1 cell line derived from *Poeciliopsis lucida*, mitochondria exhibited abnormal morphology and contained internalized copper nanoparticles [115].

Histopathological alterations represent a significant adverse effect of copper nanoparticle toxicity in aquatic organisms [116]. In fish, the damage primarily affects organs including the kidneys, liver, and gills [117,118]. Al-Bairuty et al. (2013) observed multi-organ damage in *Oncorhynchus mykiss* (liver, brain, intestines, gills, and muscle), with Cu-NPs causing more severe pathology in intestinal, cerebral, and hepatic tissues compared to CuSO_4_ [119]. The lesions included the presence of necrotic cells in the examined organs, gill edema, and detachment of blood vessel walls from the endothelium in the liver [119]. In *Carassius auratus* and *Oreochromis niloticus*, CuO-NP exposure induced gill endothelial hyperplasia (particularly in the apical and lamellar regions), cartilage tissue deformation, lamella fusion, as well as hepatic necrosis, pyknotic nuclei, edema, and the presence of blood cells within the liver tissue [120,121]. Moreover, Badran and Hamed (2024) found that chemically synthesized CuO-NPs induced more severe, dose-dependent damage compared to green-synthesized nanoparticles [120].

### 6.2. Cu-NPs Genotoxicity

Genotoxicity is one of the negative effects of nanoparticle exposure, including copper, whose mechanisms are commonly divided into primary and secondary genotoxicity [122]. Copper nanoparticles can cause primary DNA damage through indirect mechanisms, such as oxidative stress induction, which involves base pair oxidation, particularly the formation of 7,8-dihydro-8-oxoguanine (8-oxoG) [123]. Additionally, observable effects include strand breaks, crosslink formation, and chromosomal aberrations, ultimately leading to mutations [94,123]. Copper nanoparticles exhibit high affinity for electron-rich molecules, enabling direct interactions with nuclear genetic material that can cause DNA degradation and strand breaks [88,124,125]. In fish species such as *Clarias gariepinus*, *Ctenopharyngodon Idella,* or *Cyprinus carpio*, copper nanoparticles, such as CuO-NPs, induce the formation of micronuclei as well as damage to the nuclear structure in erythrocytes and gill cells [119,120,121]. No DNA degradation was observed in *Salmo trutta* hatchlings exposed to Cu-NPs at concentrations of 0.5, 1.0, 2.0, 4.0, and 8.0 mg dm^−3^ [126], while DNA fragmentation in hepatocytes was observed in *Oreochromis niloticus* at all doses tested [127]. In our studies, PCR amplification products were absent following exposure to Cu-NPs at all doses, while copper compound nanoparticles (Cu, CuO, CuZnFe_2_O_4_) showed dose-dependent inhibition [126]. Naz et al. (2020) proposed that copper nanoparticles interfere with genetic material, disrupting replication processes, a finding consistent with our experimental results [88,126]. In *Hypophthalmichthys nobilis* gametes, CuO-NPs were shown to induce concentration-dependent DNA damage [128], with similar genotoxic effects observed in adult specimens of the same species [129]. In contrast, Auclair et al. (2023) demonstrated DNA lesions in *Oncorhynchus mykiss* exposed to CuO-NPs, showing correlation with COX activity [130].

### 6.3. Gene Expression Disturbances in Fish and Their Hatchlings

Oxidative stress is one of the main toxicity mechanisms induced by copper nanoparticles, leading to excessive generation of reactive oxygen species (ROS), such as H_2_O_2_ and O_2_^•−^ [100] (Table 2). Our experimental data confirmed this phenomenon in hatchlings of *Cyprinus carpio* exposed to copper nanopowder, colloidal copper, and CuO-NPs [83]. Organisms have evolved specialized defense mechanisms to protect against the detrimental effects of free radicals. The first line of defense against oxidative stress consists of antioxidant enzymes known as free radical scavengers: glutathione peroxidase (GPx), superoxide dismutase (SOD), and catalase (CAT). GPx represents a family of selenoenzymes with multiple isoforms (varying by species), present in both animals and plants [131]. SOD, in contrast, constitutes an enzyme family comprising four distinct classes: Cu/Zn-SOD, Mn-SOD, Fe-SOD, and Ni-SOD [132,133]. GPx and SOD are primarily located in the cytosol and mitochondria [133,134], while CAT is additionally found in peroxisomes [135]. The activity of these enzymes is tightly coordinated in response to oxidative stress. Initially, superoxide anions (O_2_^•−^) generated endogenously or exogenously undergo SOD-catalyzed dismutation, resulting in the formation of hydrogen peroxide (H_2_O_2_) and molecular oxygen (O_2_). Subsequently, H_2_O_2_ is reduced to water and oxygen by GPX, using glutathione as an electron donor, or directly by CAT [136,137,138]. Current evidence suggests that GPx demonstrates higher efficiency under high intracellular ROS concentrations, whereas CAT is more effective at lower oxidative stress levels [134]. Decreased GPx activity and expression were observed in *C. carpio* hatchlings [83], while the RTG-2 cell line (derived from *Oncorhynchus mykiss* gonads) showed upregulated expression of *gpx1a*, *gpx4b*, *cat,* and *sod2* following exposure to 12.5 µg dm^−3^ [139]. In juvenile *Oreochromis niloticus*, dose-dependent upregulation of *sod*, *cat*, and *gpx* expression was observed [140]. Similarly, in our studies, increased *gpx* expression was observed in *Oncorhynchus mykiss* hatchlings exposed to CuO-NPs at concentrations of 4–256 mg dm^−3^, while *sod* expression was downregulated compared to controls [84]. Additionally, a decreased CAT activity was observed in *C. carpio* hatchlings exposed to copper nanopowder, colloidal copper, and CuO-NPs at 1 mg dm^−3^ [83]. Similarly reduced CAT activity was also recorded in *Cyprinus carpio* liver following exposure to sublethal doses of both Cu-NPs and CuO-NPs [141]. In *Dicentrarchus labrax* hatchlings, CAT activity increased after 24 h exposure to Cu-NPs at both 0.1 and 1 mg dm^−3^, while SOD activity was elevated only at the higher concentration (1 mg dm^−3^) [70]. However, in our studies on *Cyprinus carpio* hatchlings, a decrease in the activity was observed for all tested nanoparticles at this concentration [83]. These results align with observations by Canli and Canli (2020), where CAT activity in *Oreochromis niloticus* decreased at every tested dose of CuO-NPs (i.e., 1, 5, and 25 mg dm^−3^) [142]. This suggests that the activity of antioxidant enzymes may be species-dependent. Enzymatic activity is also likely organ-specific, as observed by Riaz et al. (2020) for SOD, CAT, and GST in fish, and by Villarreal et al. (2014) in their studies, where the activity of SOD and CAT decreased in the liver of *Oreochromis mossambicus* at a dose of 5 mg dm^−3^, while it increased in the gills [117,143]. In the study by Kumar et al. (2024), the activity of CAT, SOD, and GPX increased in the organs of *Pangasianodon hypophthalmus*, including the gills, liver, and kidneys [110]. In addition, in the gills and liver of *Oreochromis niloticus*, SOD and CAT activities were shown to decrease, while GPX activity increased [144].

Heat shock proteins (HSP70) are among other proteins associated with protection against oxidative stress. HSP70 belongs to a conserved protein family that, under normal physiological conditions, is responsible for maintaining protein homeostasis, including proper protein folding and degradation [145,146]. These proteins also show increased activity in response to stress stimuli such as temperature (heat shock), and oxidative or chemical stress (e.g., heavy metals) [145,146]. In fish, HSP70 activity is considered particularly critical during early developmental stages (embryonic growth and hatch), as these periods show heightened sensitivity to environmental stressors such as temperature fluctuations and heavy metal exposure [147,148]. HSP70 serves as a key protective mechanism, mitigating the damaging effects of these stressors [149]. Elevated HSP70 activity has been documented in many fish species, including *Rutilus kutum*, *Oreochromis niloticus*, and *Carassius auratus*, following exposure to various nanoparticles such as silver, zinc oxide, and aluminum oxide [150,151,152]. Similarly, an increase in the expression and activity of HSP70 was observed in *C. carpio* hatchlings exposed to copper nanopowder, colloidal copper, and CuO-NPs, as well as in *Oncorhynchus mykiss* hatchlings for all tested concentrations (4–256 mg dm^−3^), demonstrating the highest expression levels among the analyzed genes [83,84]. Supporting these findings, Abdel-Latif et al. (2021) reported elevated expression of this gene in *Oreochromis niloticus* [140]. A dose-dependent increase in both HSP70 expression and activity was also observed in the intestines of juvenile *Epinephelus coioides* exposed to Cu-NPs [114].

Cytochrome P450 enzymes (CYP) constitute a superfamily of hemoproteins responsible for xenobiotic metabolism and detoxification. They catalyze substrate oxidation (with each cytochrome showing substrate specificity) in the presence of a reducing agent [153]. In fish, 18 CYP families have been identified, including the CYP1 family, which consists of the CYP1A, CYP1B, CYP1C, and CYP1D [154,155]. There is limited literature data on studies investigating changes in *cyp1a* gene expression in aquatic organisms following exposure to copper nanoparticles. Increased *cyp1a* expression was detected in *Oncorhynchus mykiss* hatchlings [84]. On the other hand, increased gene expression but decreased enzymatic activity was observed in *Cyprinus carpio* hatchlings exposed to all tested copper nanoparticle forms [83]. An increase in the expression was observed in the gills and liver of *Oreochromis mossambicus* [143]. The literature data also demonstrate the influence of other metal nanoparticles on CYP1A expression and activity. Scown et al. (2010) reported a significant increase in *cyp1a* expression in the gills of juvenile *Oncorhynchus mykiss* exposed to 10 µg dm^−3^ Ag-NPs, while Mansour et al. (2021) observed a dose-dependent upregulation (3.31–26.50 mg dm^−3^) in *Oreochromis niloticus* [156,157]. On the other hand, the expression of *gpx*, *cyp1a*, *hsp70*, and *sod* in *Oncorhynchus mykiss* hatchlings showed no dose-dependent variation [84].

The increase in the activity and expression of antioxidant enzymes is associated with the physiological response of the organism to the presence of ROS generated as a result of oxidative stress induced by copper nanoparticles. Conversely, the subsequent decline in both enzymatic activity and gene expression suggests that antioxidant defense mechanisms are becoming overloaded by excessive ROS concentrations. Noureen et al. (2018) further suggested that this phenomenon results from inhibited synthesis of these enzymes [141].

**Table 2:** Toxic effects of Cu-NPs and CuO-NPs on various fish species.

Nanoparticle Type	Species	Disorder Type	NPs Concentration	Adverse Effects	Source
Cu-NPs	*Oncorhynchus mykiss*	Histopathological	20, 100 µg dm^−3^	Intestinal, cerebral, hepatic tissue damages	[119]
	*Epinephelus* *Coioides*	Cytotoxic	20, 100 µg dm^−3^	Mitochondrial membrane damages	[114]
	PLHC-1 cell line (derived from *Poeciliopsis lucida*)	Cytotoxic	25 µg dm^−3^	Mitochondrial damages	[115]
	*Ctenopharyngodon idella*, *Clarias gariepinus*, *Cyprinus carpio*	Genotoxic	40 mg dm^−3^, 6.25–100.00 mg dm^−3^, 2.5, 6.25, 10 mg dm^−3^	Micronuclei formation	[112,158,159]
	*Oreochromis* *niloticus*	Genotoxic	1/10, 1/20 of LC_50_ (150 mg dm^−3^)	DNA fragmentation in hepatocytes	[127]
	*Salmo trutta*	Genotoxic	1–0.0625 nM (dose-dependent)	Replication inhibition	[126] ^a^
	*Cyprinus carpio*	Molecular	1 mg dm^−3^	ROS and free radical generation	[83] ^a^
	*Cyprinus carpio*	Molecular	1 mg dm^−3^	*gpx*, *cat*, *sod* expression decrease and *cyp1a*, *hsp70* increase	[83] ^a^
	*Dicentrarchus labrax*	Molecular	0.1 (only CAT), 1 mg dm^−3^	CAT and SOD activity increase	[70]
	*Cyprinus carpio*	Molecular	1 mg dm^−3^	HSP70 enzymatic activityincrease	[83] ^a^
	*Epinephelus* *coioides*	Molecular	20, 100 µg dm^−3^	*hsp70* expression and enzymatic activityincrease (dose-dependent)	[114]
CuO-NPs	*Oncorhynchus mykiss*	Histopathological	20, 100 µg dm^−3^	Multi-organ damages, gill edema, necrotic cells	[119]
	*Carassius auratus*	Histopathological	10–100 mg dm^−3^	Gill endothelial hyperplasia, nuclei damages, hepatic necrosis	[120,121]
	*Oreochromis niloticus*	Histopathological	25, 50 mg dm^−3^	Gill endothelial hyperplasia, nuclei damages, hepatic necrosis	[120,121]
	*Apistogramma agassizii*	Cytotoxic	3 mg dm^−3^	Mitochondrial proton leakage	[113]
	*Salmo trutta*	Genotoxic	1–0.0625 nM (dose-dependent)	Replication inhibition	[126] ^a^
	*Hypophthalmichthys nobilis*	Genotoxic	2–20 mg dm^−3^ (dose-dependent)	DNA damage	[128]
	*Oncorhynchus mykiss*	Genotoxic	50 µg dm^−3^	DNA lesions	[130]
	*Cyprinus carpio*	Molecular	1 mg dm^−3^	ROS and free radical generation	[83] ^a^
	*Oreochromis* *niloticus*	Molecular	10, 20, 50 mg dm^−3^	ALP, ALT, AST, and creatinine level increase	[140]
	RTG-2 cell line (derived from *Oncorhynchus mykiss* gonads)	Molecular	12.5 µg dm^−3^	*gpx1a*, *gpx4b*, *cat,* and *sod2* expression increase	[139]
	*Oreochromis* *niloticus*	Molecular	1, 5, 25 mg dm^−3^	CAT activity decrease	[142]
	*Oreochromis* *niloticus*	Molecular	20, 50 mg dm^−3^	*sod*, *cat*, *gpx hsp70* expression increase	[140]
	*Cyprinus carpio*	Molecular	1 mg dm^−3^	*gpx*, *cat*, *sod* expressiondecrease and *cyp1a*, *hsp70* increase	[83] ^a^
	*Cyprinus carpio*	Molecular	1 mg dm^−3^	GPX, CYP1A, CAT, SOD enzymatic activitydecrease	[83] ^a^
	*Oreochromis* *mossambicus*	Molecular	5 mg dm^−3^	Liver SOD and CAT decrease	[143]
	*Oreochromis* *mossambicus*	Molecular	0.5 mg dm^−3^	*cyp1a* expression increase	[143]
	*Cyprinus carpio*	Molecular	1 mg dm^−3^	HSP70 enzymatic activityincrease	[83] ^a^
	*Oncorhynchus mykiss*	Molecular	4–256 mg dm^−3^	*gpx*, *cyp1a*, *hsp70* expression increase and *sod* decrease	[84] ^a^

References marked with “a” represent our studies.

In nanotoxicity studies, including copper nanoparticles, their adverse effects are frequently compared with ionic metal forms such as CuCl_2_ or CuSO_4_ [160,161] (Table 3). Numerous publications compared the toxicity of copper nanoparticles with that of copper sulfate (CuSO_4_), a common pollutant in aquatic environments. CuSO_4_ is widely used in aquaculture as an algaecide, herbicide, and fungicide [162], explaining its prevalent presence in aquatic environments. It is a compound with well-documented toxicity and can be applied as a useful reference point for assessing the toxicity of other copper compounds that contaminate aquatic environments, such as copper nanoparticles.

In studies on *O. mykiss* hatchlings exposed to CuSO_4_, higher expression levels compared to CuO-NPs were observed for *gpx*, *hsp70*, and *sod* at concentrations of 8–96 mg dm^−3^, and for *cyp1a* at 4, 16, 96, and 128 mg dm^−3^ [84]. For CuO-NPs, higher expression levels were recorded for *gpx* at concentrations of 128 and 256 mg dm^−3^, for *cyp1a* at 8, 64, and 128 mg dm^−3^, and for *hsp70* at 4 mg dm^−3^.

This suggests that CuSO_4_ is more toxic to the studied organism than CuO-NPs. Whether CuO-NPs cause more severe disturbances than CuSO_4_ is a subject of ongoing debate in the literature. Based on their own studies, Wang et al. (2015) concluded that in the intestines of *Epinephelus coioides*, CuO-NPs and CuSO_4_ exhibited similar toxicity, with CuO-NPs showing slightly greater potency. However, in another study of these authors on the same species, a comparable toxicity of both compounds was reported, with CuSO_4_ demonstrating a stronger effect in hepatocytes [114,163]. This suggests that the toxicity of both compounds is tissue-dependent, a finding also confirmed by Al-Bairuty et al. (2013) [119]. Wang et al. (2016) reported decreased expression and activity of both Mn-SOD and Cu/Zn-SOD after exposure to CuO-NPs and CuSO_4_ [163]. A decrease in *sod* expression was observed in *O. mykiss* hatchlings exposed to all tested doses of CuO-NPs, while an increase in the expression of this gene was recorded following exposure to CuSO_4_ [84]. In studies involving biochemical analyses (such as measurements of Na^+^/K^+^-ATPase activity), hematological parameters, and assessments of ion and glutathione levels in *Oncorhynchus mykiss*, a similar degree of toxicity was observed for both CuO-NPs and CuSO_4_ [164]. However, Isani et al. (2013) reported a higher level of DNA damage induction in *O. mykiss* exposed to CuSO_4_ compared to CuO-NPs [165]. Similarly, Soliman et al. (2021) also observed greater toxicity of CuSO_4_, recording higher genotoxicity and increased SOD and CAT activity in *Oreochromis niloticus* compared to CuO-NPs at a dose of 15 mg dm^−3^ [166].

Collectively, these studies show that although CuSO_4_ often displays higher toxicity, CuO-NPs can also cause measurable toxic effects, including oxidative stress, genotoxicity, as well as hematological and histopathological alterations. This highlights the need for further research concerning the adverse effects of copper nanoparticles and for monitoring their concentrations in aquatic environments. Thit et al. (2017) reached a similar conclusion, emphasizing that despite the lower toxicity of CuO-NPs, continuous environmental monitoring of these nanoparticles remains essential [167]. In addition to the need to protect fish embryos (such as *Danio rerio*) from the effects of CuO-NPs, Pereira et al. (2023) also highlighted differences in the exposure mechanisms between CuO-NPs and ionic copper compounds [160]. Khan et al. (2024) further emphasized that metal nanoparticles, including copper, silver, and gold oxides, should be restricted to diagnostic and therapeutic applications, contingent on a comprehensive understanding of their toxicity mechanisms [158].

**Table 3:** A comparison of CuO-NPs and Cu ions derived from CuSO_4_ toxicity, shown on *Oncorhynchus mykiss* model.

Compound Type	Species	Adverse Effects	Source
CuO-NPs	*Oncorhynchus mykiss*	*gpx*, *cyp1a*, *hsp70* expression increase and *sod* decrease	[84] ^a^
	*Oncorhynchus mykiss*	Na^+^/K^+^-ATPase activity decrease, plasma depletion *	[164]
	*Oncorhynchus mykiss*	DNA damage	[165]
CuSO_4_	*Oncorhynchus mykiss*	*gpx*, *cyp1a*, *hsp70*, *sod* * expression increase	[84] ^a^
	*Oncorhynchus mykiss*	Na^+^/K^+^-ATPase activity decrease, Na^+^ concentration decrease *	[164]

“*” next to the toxic effect indicates higher toxicity of a compound. References marked with “a” represent our studies.

### 6.4. Toxicity Towards Juvenile Fish

In addition to the effects of Cu-NPs in adult fish, it is also important to investigate their impact on juveniles at various developmental phases (including hatchlings, embryos, and eggs) (Table 4), as they are regarded as equally or even more susceptible than adults to the harmful effects of pollutants present in the aquatic environment [168].

Exposure to CuO-NPs has been shown to affect the microbiome of hatchlings, leading to physiological disorders [169]. Chorion plays a protective role, shielding the embryo from harmful environmental factors. Nanoparticles and nanomaterials, such as quantum dots and zinc nanoparticles, aggregate and deposit on the surface of the chorion, often disrupting its integrity [170,171]. CuO-NPs can also block pores in the chorion, leading to impaired hatching and increased oxidative stress [172]. Furthermore, Thit et al. (2017) reported that the deposition of CuO-NPs on the surface of *Danio rerio* embryos could lead to the release of copper ions into the embryo [167]. Chao et al. (2021) confirmed a correlation between delayed hatching in *Danio rerio* and the release of copper ions from CuO-NPs in low-ionic-strength environments. They also reported that smaller particles exerted greater cardiotoxicity than the larger ones [173]. During the swelling phase, fish eggs are particularly prone to absorbing substances, including nanoparticle pollutants, due to the increased chorion permeability [174]. Exposure of hatchlings and embryos to Cu-NPs has been shown to cause not only delayed hatching but also growth and developmental disturbances. The effects observed in *Danio rerio* exposed to CuO-NPs included notochord malformations, yolk sac swelling, and disorders of the circulatory system, such as pericardial edema and impaired cardiac function [173,175]. Additionally, Ganesan et al. (2016) reported deformities of the head, tail, and yolk sac [176]. Hatchlings were also shown to develop histopathological alterations, including liver and gill damage [177]. Studies conducted on *Cyprinus carpio* hatchlings have demonstrated increased apoptosis, while the intestines of *Epinephelus coioides* juveniles showed elevated expression and activity of enzymes associated with oxidative stress [114]. Studies on juvenile *Takifugu fasciatus* further revealed that copper nanoparticle-induced apoptosis occurred through both the caspase-dependent mitochondrial pathway and the p53-Bax-Bcl2 signaling axis [178].

**Table 4:** Selected disorders induced by copper-based nanoparticles in various fish species in different life stages. References marked in bold represent our studies.

Disorder	Nanoelement	Developmental Stage	Species	Source
Genotoxicity	Cu-NPs (nanopowder) CuO-NPs CuZnFe_2_O_4_-NPs	Hatchlings	*Salmo trutta*	[126] ^a^
Immunotoxicity	Cu-NPs	Adult form	*Takifugu fasciatus*	[178]
Hepatotoxicity	Cu-NPs	Adult form	*Takifugu fasciatus*	[179]
Gill damage	CuO-NPs Cu-NPs	Adult form	*Cyprinus carpio* *Takifugu fasciatus*	[180] [179]
Oxidative stress	Cu-NPs	Adult form	*Takifugu fasciatus*	[178]
	CuO-NPs CuO-NPs Cu-NPs (colloidal form) Cu-NPs (nanopowder)	Hatchlings	*Cyprinus carpio* *Oncorhynchus mykiss* *Cyprinus carpio* *Cyprinus carpio*	[83] ^a^, [181] [84] ^a^ [83] ^a^ [83] ^a^
Apoptosis induction	Cu-NPs CuO-NPs	Adult form Hatchlings	*Takifugu fasciatus* *Cyprinus carpio*	[178] [181]
Edema Notochord malformation Delayed hatching Bioaccumulation	CuO-NPs	Embryo Hatchlings	*Danio rerio* *Cyprinus carpio*	[173] [181]

References marked with "a" represent our studies.

## 7. Conclusions and Future Perspectives

The data presented in this review confirm that the uncontrolled release of copper-based nanoparticles poses a significant risk to aquatic organisms, particularly fish hatchlings. This highlights the necessity of controlling the release of nanoparticles, including copper, into aquatic environments. Studies monitoring Cu-based NPs concentrations in the aquatic environment should consider the ability of these nanoparticles to convert into ionic forms under certain environmental conditions (e.g., low pH), which may complicate analyses by distorting the actual concentration of Cu-based NPs in water.

It is also crucial to continue research aimed at assessing the toxicity and ecological impact of released copper nanoparticles (Cu-NPs) on aquatic ecosystems, particularly targeting protected species, farmed fish, and their hatchlings. Additionally, studies should compare the toxicity mechanisms of Cu-NPs between all developmental stages, including juvenile forms (eggs, embryos, hatchlings) and adults.

The positive effects of Cu-NPs (such as their use as biocides, catalysts, and conductors) preclude complete elimination of the threat posed by Cu-NPs in the environment. Therefore, further research should be conducted to explore less toxic alternatives to synthetic Cu-NPs or to reduce their harmful properties. These methods primarily focus on reducing the release of ions from Cu-NPs by improving their stability. The “green” synthesis of Cu-NPs is one of these methods, which utilizes plant extracts or biological systems, such as fungi, to produce nanoparticles [182]. This type of approach not only reduces toxic by-product formation during synthesis, but Cu-NPs produced during this process show potentially lower toxicity due to reduced capacity to release copper, resulting from increased particle stability [183]. Surface modifications, such as the addition of chitosan chains, polysaccharides, or carbon, have been reported to reduce Cu-NP ion release, thereby decreasing their toxicity [104,184]. Additionally, needle- or platelet-shaped Cu-NP particles exhibit higher toxicity and immunological mobilization compared to, e.g., octahedral particles [185]. Modifying nanoparticles to possess negative or neutral surface charges has been demonstrated to significantly decrease their toxicity [186]. Therefore, another approach to lowering the toxicity of Cu-NPs involves various modifications, including adjustments to their surface properties, charge, or shape.

In summary, it is essential to implement measures to control the release of Cu-NPs into the environment and to intensify research aimed at thoroughly understanding their internalization, bioaccumulation, and toxicity mechanisms.

## Data Availability

Not applicable.
